# Identification of α,β-Hydrolase Domain Containing Protein 6 as a Diacylglycerol Lipase in Neuro-2a Cells

**DOI:** 10.3389/fnmol.2019.00286

**Published:** 2019-11-26

**Authors:** Annelot C. M. van Esbroeck, Vasudev Kantae, Xinyu Di, Tom van der Wel, Hans den Dulk, Anna F. Stevens, Simar Singh, Alexander T. Bakker, Bogdan I. Florea, Nephi Stella, Herman S. Overkleeft, Thomas Hankemeier, Mario van der Stelt

**Affiliations:** ^1^Department of Molecular Physiology, Leiden Institute of Chemistry, Leiden University, Leiden, Netherlands; ^2^Department of Systems Biomedicine and Pharmacology, Leiden Academic Centre for Drug Research, Leiden University, Leiden, Netherlands; ^3^Department of Pharmacology, University of Washington, Seattle, WA, United States; ^4^Department of Psychiatry and Behavioral Sciences, University of Washington, Seattle, WA, United States; ^5^Department of Bio-Organic Synthesis, Leiden Institute of Chemistry, Leiden University, Leiden, Netherlands

**Keywords:** α, β-hydrolase domain containing protein 6, diacylglycerol lipase, 2-AG, endocannabinoids, lipidomics, chemical proteomics, activity-based protein profiling

## Abstract

The endocannabinoid 2-arachidonoylglycerol (2-AG) is involved in neuronal differentiation. This study aimed to identify the biosynthetic enzymes responsible for 2-AG production during retinoic acid (RA)-induced neurite outgrowth of Neuro-2a cells. First, we confirmed that RA stimulation of Neuro-2a cells increases 2-AG production and neurite outgrowth. The diacylglycerol lipase (DAGL) inhibitor DH376 blocked 2-AG production and reduced neuronal differentiation. Surprisingly, CRISPR/Cas9-mediated knockdown of DAGLα and DAGLβ in Neuro-2a cells did not reduce 2-AG levels, suggesting another enzyme capable of producing 2-AG in this cell line. Chemical proteomics revealed DAGLβ and α,β-hydrolase domain containing protein (ABHD6) as the only targets of DH376 in Neuro-2a cells. Biochemical, genetic and lipidomic studies demonstrated that ABHD6 possesses DAGL activity in conjunction with its previously reported monoacylglycerol lipase activity. RA treatment of Neuro-2a cells increased by three-fold the amount of active ABHD6. Our study shows that ABHD6 exhibits significant DAG lipase activity in Neuro-2a cells in addition to its known MAG lipase activity and suggest it is involved in neuronal differentiation.

## Introduction

**FIGURE 1:**
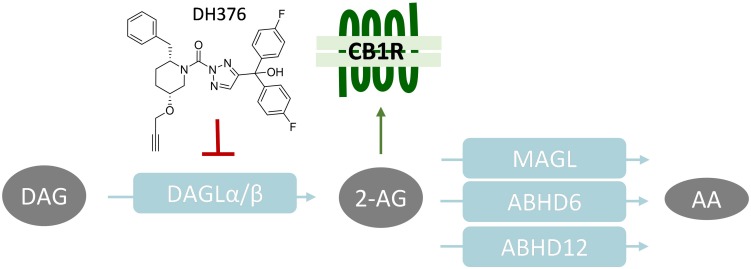
Schematic overview of 2-AG signaling and metabolism. CB1R, Cannabinoid receptor type 1; DAGL, diacylglycerol lipase; MAGL, monoacylglycerol lipase; ABHD, α,β-hydrolase domain containing protein; DAG, diacylglycerol; 2-AG, 2-arachidonoylglycerol; AA, arachidonic acid.

The endocannabinoid 2-archidonoylglycerol (2-AG) is an important signaling lipid in the central nervous system (CNS). It acts as a retrograde messenger that activates the presynaptic cannabinoid receptor type 1 (CB1R), thereby regulating neurotransmitter release. 2-AG is involved in a variety of physiological processes, including modulation of memory, energy balance, and emotional states, such as stress and anxiety (Di Marzo, 2011). Biochemical, pharmacological and genetic studies have established diacylglycerol lipases α and β (DAGLα, DAGLβ) as the main biosynthetic enzymes that produce 2-AG in the brain by catalyzing the *sn*-1-specific hydrolysis of diacylglycerol (DAG) to generate 2-AG (Bisogno et al., 2003; Figure 1). For example, congenital deletion of DAGLα or DAGLβ resulted in 80 and 50% reduction, respectively, of brain 2-AG levels in knockout (KO) mice as compared to wild type (WT) littermates (Gao et al., 2010). Pharmacological studies with covalent, irreversible, dual DAGL inhibitors, such as DH376 and DO34, showed that acute blockade of 2-AG biosynthesis in the mouse brain reduced neuroinflammatory responses (Ogasawara et al., 2016), reversed LPS-induced anapyrexia (Ogasawara et al., 2016), reduced food intake (Deng et al., 2017), and modulated cocaine-seeking behavior (McReynolds et al., 2018) and stress responses (Bluett et al., 2017).

The life span of 2-AG signaling at the synapse is tightly controlled. Monoacylglycerol lipase (MAGL) (Dinh et al., 2002) and α,β-hydrolase domain containing protein 6 and 12 (ABHD6, ABHD12) have been identified as the key enzymes terminating the physiological role of 2-AG. They hydrolyze the ester bond in 2-AG, thereby generating glycerol and arachidonic acid (AA) (Blankman et al., 2007; Marrs et al., 2010; Figure 1). MAGL is the predominant lipase in the brain covering over 85% of 2-AG hydrolysis, whereas ABHD6 and ABHD12 account for 4 and 9%, respectively (Blankman et al., 2007).

2-Archidonoylglycerol signaling is not only important in the adult brain, multiple studies have also provided evidence of a functional role of 2-AG during neural developmental processes (Oudin et al., 2011b), including axonal growth and guidance (Bisogno et al., 2003; Harkany et al., 2007; Watson et al., 2008; Wu et al., 2010), differentiation (Jung et al., 2011), and neurogenesis (Gao et al., 2010; Oudin et al., 2011a). In adult mice, DAGLα is mainly restricted to postsynaptic sites on neurons, whereas DAGLβ is expressed in microglial cells. Importantly, DAGLα and DAGLβ are expressed by neurons at developing axonal tracts during neuronal development (Bisogno et al., 2003; Berghuis et al., 2007; Watson et al., 2008; Oudin et al., 2011a). Jung et al. (2011) have investigated the role of DAGLs in neuronal differentiation using retinoic acid (RA)-induced neurite outgrowth in murine neuroblastoma cell line Neuro-2a and found that RA elevated cellular 2-AG levels in Neuro-2a cells during differentiation and recombinant expression of DAGLα or DAGLβ increased neurite outgrowth, whereas silencing the expression of DAGLs using shRNAs reduced the number of cells with neurites. The contribution of the endogenously expressed DAGL enzymes to 2-AG biosynthesis in these cells is, however, less clear. Here, we sought to further test the role of the two DAGL isoforms in 2-AG biosynthesis in Neuro-2a using pharmacological, analytical, and genetic methods.

## Materials and Methods

### Materials, Probes, and Inhibitors

Fluorophosphonate-rhodamine (FP-TAMRA) was purchased from Thermo Fisher, as well as synthesized in-house as previously described (Janssen et al., 2018). FP-Biotin was purchased from Santa Cruz Biotechnology, KT182 was purchased from Sigma Aldrich. Fluorophosphonate-BODIPY (FP-BODIPY) (Janssen et al., 2018), MB064 (Baggelaar et al., 2013), MB108 (Baggelaar et al., 2013), DH376 (Ogasawara et al., 2016), and LEI105 (Baggelaar et al., 2013) were synthesized as previously described. All synthesized compounds were at least 95% pure as analyzed by LC-MS, NMR, and HRMS. Primers were ordered from Sigma Aldrich or Integrated DNA Technologies. Other chemicals, reagents were purchased from Sigma Aldrich, unless indicated otherwise.

### Cloning General

Full-length human DAGLα and ABHD6 cDNA (Source Bioscience) was cloned into the mammalian expression vector pcDNA3.1, containing ampicillin and neomycin resistance genes. The inserts were cloned in frame with a C-terminal FLAG-tag and site-directed mutagenesis was used to generate the catalytically inactive DAGLα^S472A^ and ABHD6^S148A^ mutants. pcDNA3.1 containing the gene for eGFP was used as a transfection control. Plasmids were isolated from transformed XL-10 Z-competent cells (Midi/Maxi Prep, Qiagen), sequenced and verified (CLC Main Workbench).

### Cell Culture

#### General

Neuro-2a (murine neuroblastoma) and HEK293-T (human embryonic kidney) cells were cultured at 37°C and 7% CO_2_ in DMEM containing phenol red, stable glutamine, newborn bovine serum (10% v/v; Thermo Fisher), and penicillin and streptomycin (200 μg/mL each; Duchefa). Medium was replaced every 2–3 days and cells were passaged twice a week at ∼90% confluence by resuspension in fresh medium. Cell lines were from ATCC and were regularly tested for mycoplasma contamination. Cultures were discarded after 2–3 months of use.

#### Single Cell Clone Generation

Single cell clones of Neuro-2a cells were generated by seeding cells at a density of 0.5, 1, 2, or 4 cells per well in 96-wells plates. After several days, wells plates were screened for growth of single cell clones by phase-contrast microscopy (EVOS Auto FL2). Single cell clones were selected and expanded to full cultures.

#### Transient Transfections (HEK293-T)

One day prior to transfection, HEK293-T cells were seeded at 1 × 10^6^ cells/well in 6-wells plates or at 0.3 × 10^6^ cells/well in 12-wells plates. Prior to transfection, culture medium was aspirated and a minimal amount of complete medium was added. A 3:1 (m/m) mixture of polyethyleneimine (PEI) and plasmid DNA (1.25 μg in 6-well, 0.625 μg in 12-well) was prepared in serum-free culture medium and incubated for 15 min at rt. Transfection was performed by dropwise addition of the PEI/DNA mixture to the cells. Transfection with pcDNA3.1 encoding GFP was used to generate control samples. Twenty-four hours post-transfection, culture medium was replaced. *In situ* treatments were initiated 48 h post-transfection. Transfection efficiency was checked by fluorescence microscopy on eGFP transfected samples (EVOS FL2 Auto, GFP-channel).

#### *In situ* Treatments

The term *in situ* is used to designate experiments in which live cell cultures are treated. Neuro-2a cells were seeded at 0.3 × 10^6^ cells/well in 12-wells plates, 2.5 × 10^6^ cells/dish in 6 cm dishes, 48 h prior to treatment. HEK293-T cells from transient transfections were used at 24–48 h. post-transfection. Culture medium was aspirated and after a careful PBS wash, treatment medium (serum-free DMEM) containing vehicle (0.1% DMSO) or DH376 (100 nM – 1 μM as indicated in figure legends) was added. After incubation for 2 h at 37°C and 7% CO_2_, treatment medium was aspirated, and cells were rinsed with PBS. Subsequently cells were harvested by resuspension in PBS and pelleted (1000 *g*, 3 min, rt). Cell pellets were flash frozen in liquid nitrogen and stored at −80°C until further use.

#### Retinoic Acid Stimulation

**FIGURE 2:**
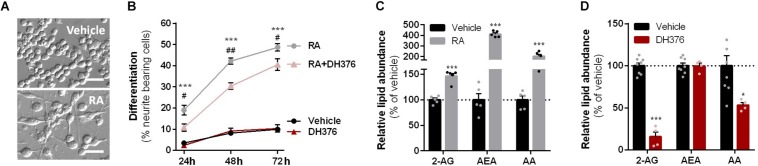
RA-induced differentiation increased cellular 2-AG levels and is reduced by DH376 treatment. **(A–C)** Neuro-2a cells were differentiated with RA (50 μM, 2% serum, 24–72 h) in the presence or absence of inhibitor DH376 (100 nM). **(A)** Phase contrast microscopy of representative differentiated and non-differentiated Neuro-2a cultures (72 h incubation). Scale bar: 50 μm. **(B)** Neuro-2a differentiation was quantified as the percentage of neurite bearing cells [mean ± SEM (*n* = 3), *t*-test: ^∗∗∗^*p* < 0.001 vehicle versus RA, ^#^*p* < 0.05, ^##^*p* < 0.01 RA versus RA-DH376]. **(C)** Lipidomics analysis on vehicle and RA-stimulated cells (72 h). Lipid abundance was normalized to the number of cells. Data is expressed as % of vehicle [mean ± SEM (*n* = 5), *t*-test: ^∗∗∗^*p* < 0.001]. **(D)** Lipidomics analysis on *in situ* DH376-treated Neuro-2a (100 nM, 2 h). Lipid abundance was normalized for the amount of protein. Data is expressed as % of vehicle [mean ± SEM (Veh: *n* = 8, DH376: *n* = 4), *t*-test: ^∗^*p* < 0.05, ^∗∗∗^*p* < 0.001].

Neuro-2a cells were seeded at 1 × 10^5^ cells/well in 6-well plates or 1 × 10^6^ cells/dish in 10 cm dishes. One day after seeding, medium was aspirated and RA stimulation was initiated by adding DMEM containing 2% newborn bovine serum and all-*trans*-retinoic acid (50 μM) or vehicle (0.1% DMSO). For Figure 2D, co-treatment was done with vehicle (0.1% DMSO) or DH376 (100 nM) throughout the entire differentiation process. Neurite outgrowth was investigated after 24, 48 or 72 h using phase contrast microscopy (Olympus or EVOS FL2 Auto, phase contrast, large ring). Neurite outgrowth was quantified by counting the cells with a minimum of two outgrowth processes longer than the cell body, as a percentage of the total number of cells (three dishes, five images per dish). Cell count and viability were checked by Trypan blue staining and automated cell counting (TC20^TM^ Cell Counter, Bio-Rad).

### CRISPR/Cas9-Mediated Knockdowns

#### Guide Design and Constructs

Two sgRNA’s, in early exons, with high efficiency and specificity as predicted by CHOPCHOP v2 online web tool^1^ (Labun et al., 2016) were selected. Guides were cloned into the *Bbs*I restriction site of plasmid px330-U6-Chimeric_BB-CBh-hSpCas9 (gift from Feng Zhang, Addgene plasmid #42230) as previously described (Cong et al., 2013; Ran et al., 2013). Constructs and primers are annotated in Table 1.

**TABLE 1:** sgRNA targets, sgRNA oligos (top, bottom) and T7E1 primers (forward, reverse).

**sgRNA target**	**#**	**Primer sequences**
*Dagla Exon* 2	447^∗^	Top: CACCGAGGATTACAAACCTGCAGAG Bottom: AAACCTCTGCAGGTTTGTAATCCTC Forward: GAACTTGGGGTCTTTTTGTCTG Reverse: CAAGGAAGAACAGGTAACCAGG
*Exon* 3	485	Top: CACCGCATGGCTGGCAGCTCTGGG Bottom: AAACCCCAGAGCTGCCAGCCATGC Forward: GGTAGTAGTTACTGCCGATGCC Reverse: CTCTTCAGGGCTGACTCAGTTT
*Daglb Exon* 1	449^∗^	Top: CACCGTGGGAGGTGCGCCATGCCG Bottom: AAACCGGCATGGCGCACCTCCCAC Forward: TTAAACAGAAATGACCACACCG Reverse: CCTGGTTTCTATGAATTGCTCC
*Exon* 2	450	Top: CACCGTGTATCTCACGCACAGAAGG Bottom: AAACCCTTCTGTGCGTGAGATACAC Forward: CTCCTACATCTCTTGCTTGCCT Reverse: ACACAAATGGTAGCGCAGTATG
*Abhd6 Exon* 2	724	Top: CACCGGTTAACATGTTTGTGATTG Bottom: AAACCAATCACAAACATGTTAACC Forward: GATCCATGGTATACCCCTAACCACTGAGTCATCTC Reverse: TGACTCGAGATTGGAATGGCGATATGGTTACACT
*Exon* 3	725^∗^	Top: CACCAGTTCGCTACGCACACCATG Bottom: AAACCATGGTGTGCGTAGCGAACT Forward: TCCAAGCTTATGCCTGCTTGTTTGCTTTTATTT Reverse: CAACACCGGTATCCTATGTTAGCTCACTCCCACCC

*Constructs indicated with an asterisk (^∗^) were used to generate double and triple knockdowns.*

#### Knockdown Population Generation

Neuro-2a cells were transfected three times (once every 3 days) to yield populations with a high knockdown efficiency. Cells were seeded at days 0, 3, and 6 and transfected at days 1, 4, and 7. Samples for T7E1 assays, and for ABPP were harvested at days 2, 5, and 11 and after additional several weeks of cell culture. One day prior to the first transfection, Neuro-2a cells were seeded to a 6-well plate to reach 80% confluence at the time of transfection. Prior to transfection, culture medium was aspirated and 2 mL of fresh medium was added. A 5:1 (m/m) mixture of PEI (17.5 μg per well) and plasmid DNA (total 3.5 μg per well) was prepared in serum-free culture medium (250 μL each) and incubated (15 min, rt). Transfection was performed by dropwise addition of the PEI/DNA mixture to the cells. Twenty-four hours post-transfection, the culture medium was replaced, a small number of cells was harvested for analysis by T7E1 assay and ABPP, while the remainder of the cells was kept in culture under standard conditions for following transfections. After three transfection rounds, the cells were cultured according to standard protocol. Aliquots of knockdown cell populations were prepared in complete DMEM +10% DMSO and stored at −150°C. Efficiency of knockdown was verified over time. Cells were discarded after 3 months of culture.

#### T7E1 Assay

Genomic DNA was obtained by mixing 50 μL QuickExtract^TM^ (Epicentre) with cell pellet (∼10% of a well from a 6-well plate). The samples were incubated at 65°C for 6 min, mixed and incubated at 98°C for 2 min. Genomic DNA extracts were diluted in sterile water and directly used in PCR reactions. Genomic PCR reactions were performed on 2.5–5 μL isolated genomic DNA extract using Phusion High-Fidelity DNA Polymerase (Thermo Fisher) in Phusion HF buffer Green (Thermo Fisher) in a final volume of 20 μL, for primers see Table 1.

For the T7E1 assay, genomic PCR products were denatured and reannealed in a thermocycler using the following program: 5 min at 95°C, 95 to 85°C using a ramp rate of −2°C/s, 85 to 25°C using a ramp rate of −0.2°C/s. Annealed PCR product (8.5 μL) was mixed with NEB2 buffer (1 μL) and T7 endonuclease I (5 U, 0.5 μL; New England Biolabs), followed by a 30 min incubation at 37°C. Digested PCR products were analyzed using agarose gel electrophoresis with ethidium bromide staining. A sample without T7 endonuclease I was also analyzed as control. Agarose gels were analyzed using Image Lab^TM^ Software (Bio-Rad) and DNA modification efficiency was expressed as percentage T7E cleavage (volume integral of digested bands/volume integral all bands × 100%). Uncropped images can be found in Supplementary Figure S6.

#### Cell Lysate Preparation

Cells were harvested in PBS and pelleted by centrifugation (1000 × *g*, 3–5 min, rt). Cell pellets were snap-frozen and stored at −80°C until further use. Cell pellets were thawed on ice, resuspended in cold lysis buffer (20 mM HEPES pH 7.2, 2 mM DTT, 250 mM sucrose, 1 mM MgCl_2_, 2.5 U/mL benzonase) and incubated on ice (15–30 min). Protein concentrations were determined by a Quick Start^TM^ Bradford Protein Assay (Bio-Rad). After dilution to 2 mg/mL in sucrose lysis buffer or storage buffer (20 mM HEPES pH 7.2, 2 mM DTT), samples were used or flash frozen in liquid nitrogen and stored at −80°C until further use. DTT was left out of all buffers for samples intended for click-chemistry.

#### Tissue Lysate Preparation

Mice were housed in a pathogenic-free facility in accordance with the National Institutes of Health; the Institutional Animal Care and Use Committee at the University of Washington approved all experiments. Flash frozen whole brain tissues (excluding cerebellum) were obtained from wild-type (male, 10 weeks) and *Abhd6*^–/–^ mice (male, 18 weeks). *Abhd6*^–/–^ mice were generated in by a targeting construct designed to delete exon 5 of the Abhd6 gene, which includes the predicted catalytic serine S148 contained within a canonical GXSXG motif, and led to absence in ABHD6 expression and activity (Deng et al., manuscript submitted). Frozen tissues were thawed on ice and dounce-homogenized in cold lysis buffer (20 mM HEPES pH 7.2, 2 mM DTT, 250 mM sucrose, 1 mM MgCl_2_, 2.5 U/mL benzonase). After incubation on ice for 15 min, tissue debris was pelleted by two sequential centrifugation steps (2500 × *g*, 3 min, 4°C). Soluble lysates were diluted to 2 mg/mL in storage buffer (20 mM HEPES pH 7.2, 2 mM DTT), flash-frozen in aliquots and stored at −80°C until further use.

### Activity-Based Protein Profiling

#### Gel-Based ABPP: Single Probe

Whole lysate (2 mg/mL) was incubated with activity-based probes MB064 (250 nM – 2 μM, 20 min, rt) or FP-TAMRA (500 nM, 20 min, rt). The reaction was quenched with Laemmli buffer (30 min, rt) and 20 μg protein was resolved by SDS-PAGE (10% acrylamide gel) along with protein marker (PageRuler^TM^ Plus, Thermo Fisher). In-gel fluorescence was detected in the Cy3- and Cy5-channel on a ChemiDoc^TM^ MP imaging system (Bio-Rad) and gels were stained with coomassie after scanning. Fluorescence was quantified and normalized to coomassie staining using ImageLab^TM^ software (Bio-Rad) and data was processed in Excel (Microsoft) and GraphPad Prism 7 (GraphPad). Uncropped images can be found in Supplementary Figure S6.

#### Gel-Based ABPP: Probe Mixture

Whole lysates (DTT-free, 2 mg/mL) were incubated with activity-based probe MB064 (2 μM, 10 min, rt), followed by incubation with FP-TAMRA (500 nM, 10 min, rt) and a subsequent conjugation to Cy5-azide by incubation with click-mix (2.5/10 μM Cy5-N3, 67 mM sodium ascorbate, 4 mM CuSO_4_(H_2_O)_5_, 1.3 mM THPTA; 30 min, rt). The reaction was quenched with Laemmli buffer (30 min, rt) and 15 μg protein was resolved by SDS-PAGE (10% acrylamide gel) along with protein marker (PageRuler^TM^ Plus, Thermo Fisher). In-gel fluorescence was detected in the Cy3- and Cy5-channel on a ChemiDoc^TM^ MP imaging system (Bio-Rad) and gels were stained with coomassie after scanning. Fluorescence was quantified and normalized to coomassie staining using ImageLab^TM^ software (Bio-Rad) and data was processed in Excel (Microsoft) and GraphPad Prism 7 (GraphPad). Uncropped images can be found in Supplementary Figure S6.

#### Chemical Proteomics With Label-Free Quantification

The chemical proteomics workflow was modified from a previously published protocol (van Rooden et al., 2018). In short, for general profiling of the serine hydrolases the whole lysates (250 μg protein, *n* = 4) were incubated with serine hydrolase probe cocktail (10 μM MB108, 10 μM FP-Biotin, 30 min, 37°C, 300 rpm). Denatured protein samples (1% SDS, 5 min, 100°C) were also analyzed as a negative control. For DH376 target identification, the whole lysates of DH376 treated cells (250 μg protein, *n* = 4) were conjugated to biotin-azide by the addition of 10× concentrated click mix (final: 1 mM CuSO_4_(H_2_O)_5_, 0.56 mM sodium ascorbate, 0.2 mM THPTA, 0.04 mM biotin-azide in MilliQ) and subsequent incubation (60 min, 37°C, 300 rpm). A vehicle treated sample was also analyzed as a negative control. Precipitation, alkylation, avidin enrichment, on-bead digestion and sample preparation was performed as described (van Rooden et al., 2018). Dried peptides were stored at −20°C until LC-MS analysis. Prior to measurement, samples were reconstituted in 50 μL LC-MS solution and transferred to LC-MS vials. Analysis was performed using Progenesis QIP (Waters) as published, using the following cut-offs: ≥2-fold enrichment compared to negative control, ≥2 peptides, ≥1 unique peptide, peptide ion correlations ≥0.7. Peptide lists are provided in Supplementary Table S1.

### Lipidomics

#### Sample Preparation: Neuro-2a Retinoic Acid Stimulation

Neuro-2a cells were seeded at 0.75 × 10^6^ cells/10 cm dish). One day after seeding, medium was aspirated and RA stimulation was initiated by adding DMEM containing 2% serum and RA (50 μM) or vehicle (0.1% DMSO). After 48–72 h (as indicated in figure legends) neurite outgrowth was assessed using phase contrast microscopy (Olympus). Cells were washed with PBS and harvested by resuspension in PBS (for RA stimulated cells, five dishes were combined to yield enough cells). Cells were pelleted (200 *g*, 10 min, rt) and resuspended in 1 mL PBS. Cell count and viability were checked by Trypan blue staining and automated cell counting (TC20^TM^ Cell Counter, Bio-Rad) and 1 or 2 × 10^6^ cells were pelleted (1000 *g*, 3 min, rt). Pellets were flash frozen in liquid nitrogen and stored at −80°C until lipid extraction.

#### Sample Preparation: Neuro-2a Single Cell Clones

Neuro-2a cells were seeded at 1.25 × 10^6^ cells/dish in a 10 cm dish. One day after seeding, medium was aspirated and cells were cultured in 2% serum and vehicle (0.1% DMSO). After 48 h, cells were washed with PBS and harvested by resuspension in PBS. Cells were pelleted (200 *g*, 5 min, rt) and resuspended in PBS. Cell count and viability were measured by Trypan blue staining and automated cell counting (TC20^TM^ Cell Counter, Bio-Rad) and 1 × 10^6^ cells were pelleted (1000 *g*, 3 min, rt). Pellets were flash frozen in liquid nitrogen and stored at −80°C until lipid extraction.

#### Sample Preparation: Neuro-2a Knockdown Populations

Neuro-2a cells were seeded at 2.5 × 10^6^ cells/dish in a 6 cm dish 48 h prior to treatment. Alternatively, HEK293-T cells from transient transfections were used at 48 h post-transfection (6-wells format). Culture medium was aspirated and after a PBS wash, treatment medium (serum-free DMEM) containing vehicle (0.1% DMSO) or DH376 (100 nM) was added. After incubation for 2 h at 37°C and 7% CO_2_, treatment medium was aspirated, and cells were washed with PBS. Subsequently cells were harvested by resuspension in 1250 μL PBS. Cell count and viability were measured by Trypan blue staining and automated cell counting (TC20^TM^ Cell Counter, Bio-Rad). Cells from 1000 μL suspension were spun down (1000 × *g*, 3 min, rt) in a low binding Eppendorf tube. Pellets were flash frozen in liquid nitrogen and stored at −80°C until lipid extraction. The remaining cell suspension (∼200 μL) was flash frozen and used to determine the protein concentration of each sample. The suspension was thawed on ice and cells were lysed by sonication using a probe sonicator (Heidolph; 5 s per sample, 10% amplitude). Protein concentrations (∼1 mg/mL) were determined by a Quick Start^TM^ Bradford Protein Assay (Bio-Rad) and were used for normalization of the lipid abundance.

#### Lipid Extraction

Lipid extraction was performed as previously described (Van Esbroeck et al., 2017) with minor adaptations. In brief, cell pellets were transferred into 1.5 mL Eppendorf tubes, spiked with 10 μL of deuterated internal standard mix (Table 2), followed by addition of 0.5% NaCl and later 100 μL of ammonium acetate buffer (0.1 M, pH 4) was added. After addition of 1000 μL methyl *tert*-butyl ether (MTBE), the tubes were thoroughly mixed for 5 min using a bullet blender (Next Advance) at medium speed, followed by a centrifugation step (16,000 × *g*, 5 min, 4°C). Then 850 μL of the upper MTBE layer was transferred to clean 1.5 mL Eppendorf tubes. Samples were dried in a SpeedVac (Eppendorf) followed by reconstitution in 50 μL of acetonitrile:water (90:10, v/v). The reconstituted samples were centrifuged (16,000 × *g*, 3 min, 4°C) before transferring into LC-MS vials. 5 μL of each sample was injected into the LC-MS/MS system.

**TABLE 2:** LC-MS standards and internal standards for lipidomics analysis.

**Standards**				
**Abbreviation**	**Metabolite**	**Q1**	**Q3**	**Polarity**
DAG (16:0, 20:4)	1-Palmitoyl-2-arachidonoyl-sn-glycerol	634	313	+
1&2-AG	2&1-Arachidonoylglycerol (20:4)	379	287	+
AEA	Anandamide (20:4)	348	62	+
DHEA	*N*-Docosahexaenoylethanolamide (22:6)	372	62	+
LEA	*N*-Linoleoylethanolamide (18:2)	324	62	+
NADA	*N*-Arachidonoyl dopamine (28:4)	440	137	+
OEA	*N*-Oleoylethanolamide (18:1)	326	62	+
PEA	*N*-Palmitoylethanolamide (16:0)	300	62	+
SEA	*N*-Stearoylethanolamide (18:0)	328	62	+
2-AGE	2-Arachidonyl glycerol ether (20:4)	365	273	+
DEA	*N*-Docosatetraenoylethanolamide (22:4)	376	62	+
DGLEA	Dihomo-γ-Linolenoyl Ethanolamide (18:3)	350	62	+
O-AEA	*O*-Arachidonoyl ethanolamine (20:4)	348	62	+
2-LG	2-Linoleoyl glycerol (18:2)	355	263	+
1-LG	1-Linoleoyl glycerol (18:2)	355	263	+
2-OG	2-Oleoyl glycerol (18:1)	357	265	+
EPEA	Eicosapentaenoyl ethanolamide (20:5)	346	62	+
POEA	*N*-Palmitoleoylethanolamide (16:1)	298	62	+
ETAEA	Eicosatrienoic acid ethanolamide (20:3)	350	62	+
PDEA	*N*-Pentadecanoyl ethanolamide (15:0)	286	62	+
α-LEA	*N*-α-Linolenylethanolamide (18:2)	322	62	+
OA	Oleic acid (18:1)	281	263	–
LA	Linoleic acid (18:2-ω6)	279	261	–
α-LA	α-Linolenic acid (18:3-ω3)	277	233	–
γ-LA	γ-Linolenic acid (18:3-ω6)	277	233	–
DGLA	Dihomo-γ-linolenic acid (20:3-ω6)	305	261	–
MA	Mead acid (20:3-ω9)	305	261	–
AA	Arachidonic Acid (20:4-ω6)	303	259	–
EPA	Eicosapentaenoic acid (20:5-ω3)	301	257	–
DTA	Docosatetraenoic acid (22:4-ω6)	332	288	–
DHA	Docosahexaenoic acid (22:6-ω3)	327	283	–

**Internal standards**				

DAG (34:0)	1-margaroyl-2-margaroyl-sn-glycerol	614	327	+
2-AG (20:4)-d8	2-Arachidonoylglycerol-d8	387	294	+
PEA (16:0)-d4	Palmitoyl ethanolamide-d4	304	62	+
SEA (18:0)-d3	Stearoyl ethanolamide-d3	331	62	+
OEA (18:1)-d4	Oleoyl ethanolamide-d4	330	66	+
LEA (18:2)-d4	Linoleoyl ethanolamide-d4	328	66	+
AEA (20:4)-d8	Arachidonoyl ethanolamide-d8	356	62	+
DHEA (22:6)-d4	Docosahexaenoyl ethanolamide-d4	376	66	+
NADA (28:4)-d8	*N*-Arachidonoyl dopamine-d8	448	137	+

#### LC-MS/MS Analysis

LC-MS/MS analysis was performed as previously described (Kantae et al., 2017; Van Esbroeck et al., 2017) with minor adaptations. A targeted analysis of 31 compounds, including endocannabinoids and related *N*-acylethanolamines (NAEs) and free fatty acids (Table 2), was detected using an Acquity UPLC I class Binary solvent manager pump (Waters) in conjugation with AB SCIEX 6500 quadrupole-ion trap (AB Sciex). The separation was performed with an Acquity HSS T3 column (2.1 × 100 mm, 1.8 μm) maintained at 45°C. The aqueous mobile phase A consisted of 2 mM ammonium formate and 10 mM formic acid, and the organic mobile phase B was acetonitrile. The flow rate was set to 0.55 mL/min; initial gradient conditions were 55% B held for 2 min and linearly ramped to 100% B over 6 min and held for 2 min; after 10 s the system returned to initial conditions and held 2 min before next injection. Electrospray ionization-MS and a selective Multiple Reaction Mode (sMRM) was used for endocannabinoid quantification. Individually optimized MRM transitions using their synthetic standards for target compounds and internal standards are described in Table 2.

#### DAG Analysis

LC-MS/MS analysis of DAG (16:0, 20:4) was performed as described in the above section with the following adaptations. The mobile phase A was 10 mM ammonium formate and 10 mM formic acid in 60:40 (v/v%) acetonitrile:water, the mobile phase B was 10 mM ammonium formate and 10 mM formic acid in 10:90 (v/v%) acetonitrile:isopropanol. The flow rate was set to 0.4 mL/min; initial gradient conditions were 50% B for 0.5 min and linearly ramped to 60% B at 2 min, then ramped to 90% B at 6 min; after 6 s the system returned to initial conditions and held 1.4 min before next injection.

### NBD-HPTLC Assay

Whole lysates of HEK293-T transiently expressing eGFP (control), DAGLα, ABHD6 or their catalytically inactive serine mutants were prepared as described above. Lysate (100 μg protein) was mixed with 5 μM DAG-NBD (Cayman Chemical; 2 mM stock in EtOH) in HEPES buffer (20 mM HEPES pH7.2, 2 mM DTT) and incubated (30 min, 37°C, 600 rpm, dark). As a control, a sample without protein was also analyzed. After incubation, lipids were extracted by a Bligh and Dyer extraction. In short, 800 μL chloroform:methanol (1:1, v/v) and 110 μL MilliQ were added to the sample. Phases were separated by centrifugation (5 min, 13,000 × *g*) and the bottom layer was transferred to a dark Eppendorf tube. The upper layer was extracted once more by adding 400 μL chloroform. The lipid extract was dried in a SpeedVac (Eppendorf) (45 min, 45°C). Lipids were reconstituted in 40 μL methanol, and lipids (2 μL, *n* = 3) were separated by thin layer chromatography on high performance thin layer chromatography (HPTLC) Silica gel 60 plates (Merck) using chloroform:methanol (80:20, v/v) as eluent. NBD-labeled lipids were detected using a Typhoon Imaging system (GE Healthcare Bio-Science) (Alexa488 channel, 250V). Fluorescence was quantified using ImageLab^TM^ software (Bio-Rad). Excel (Microsoft) and GraphPad Prism 7 (GraphPad) were used for further analysis. DAG-NBD was expressed as fraction of the total NBD intensity in each lane and normalized to eGFP samples.

NBD-HPTLC assays on tissue lysates were performed as described above with the following modifications. Tissue lysate was pre-incubated with vehicle (DMSO) or inhibitor (100 nM, 30 min, 37°C). DAG-NBD was used at with 10 μM and incubated for 15 min (37°C, 600 rpm, dark).

### Western Blot

Cell lysates were denatured with Laemmli buffer (30 min, rt) and 20 μg lysate was resolved on a 10% acrylamide SDS-PAGE gel along with PageRuler^TM^ Plus Protein Marker (Thermo Scientific). Proteins were transferred to 0.2 μm polyvinylidene difluoride membranes by Trans-Blot Turbo^TM^ Transfer system (Bio-Rad). Membranes were washed with TBS (50 mM Tris, 150 mM NaCl) and blocked with 5% milk in TBS-T (50 mM Tris, 150 mM NaCl, 0.05% Tween 20) (1 h, rt). Membranes were then incubated with primary antibody mouse-anti-FLAG (F3156, Sigma Aldrich; 1:2500 in 5% milk in TBS-T, 45 min, rt) washed with TBS-T, incubated with secondary donkey-anti-mouse-Alexa647 (A-31571, Thermo Fisher; 1:10000 in 5% milk TBS-T, 45 min, rt), and washed with TBS-T and TBS. Fluorescence was detected on the ChemiDoc^TM^ MP imaging system (Bio-Rad) in the Alexa647 channel, and Cy3/Cy5 channels for the protein marker. Signal was normalized to coomassie staining using ImageLab^TM^ software (Bio-Rad) and data was processed in Excel (Microsoft) and GraphPad Prism 7 (GraphPad).

### Statistical Methods

All statistical analyses and methods are included in the respective figure or table captions. In brief: all data are shown as the mean ± SEM, unless indicated otherwise. A Student’s *t*-test (two-tailed, unpaired) was used to determine statistical significance, with a Holm–Sidak multi-comparison correction for proteomics data using GraphPad Prism 7 (GraphPad). Samples were compared to WT/Vehicle/GFP controls and statistical significance is indicated as ^∗^*p* < 0.05, ^∗∗^*p* < 0.01, ^∗∗∗^*p* < 0.001.

## Results

To investigate the role of DAGL in neuronal differentiation, Neuro-2a cells were incubated with RA, which induced a time-dependent outgrowth of neurites (Figures 2A,B). This resulted in increased 2-AG net levels as determined by liquid chromatography-mass spectrometry (LC-MS) (Figure 2C). These findings confirm and extend a previous study (Jung et al., 2011). Of note, anandamide (AEA) and AA levels were also significantly increased (Figure 2C). To check whether endogenously expressed DAGLs are responsible for the 2-AG production during differentiation, the cells were incubated with the dual DAGL inhibitor DH376. 2-AG and AA, but not AEA, levels were reduced by DH376 (Figure 2D). The inhibitor also impaired differentiation of Neuro-2a cells, as indicated by the reduced fraction of neurite bearing cells after 24, 48, and 72 h of RA stimulation (Figure 2B). This suggested that DAGL-dependent 2-AG and/or AA production plays a role in the differentiation process, as previously suggested (Jung et al., 2011).

**FIGURE 3:**
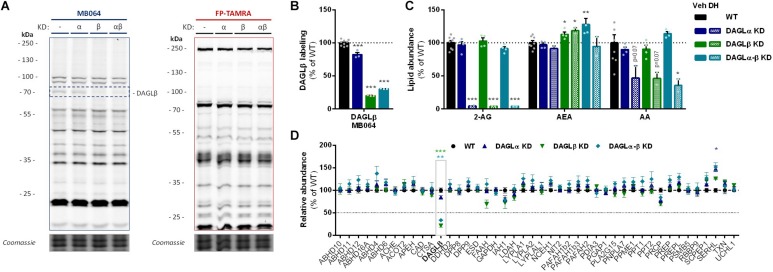
DAGL KD does not alter 2-AG levels in Neuro-2a. **(A,B)** DAGL KD populations were analyzed by gel-based ABPP using probes MB064 (2 μM) and FP-TAMRA (500 nM) (20 min, rt). Coomassie served as protein loading control. **(B)** Probe labeling of DAGLβ was quantified and normalized for protein loading. Data is expressed as % of vehicle [mean ± SEM (WT: *n* = 9, KD: *n* = 3), *t*-test: ^∗∗∗^*p* < 0.001]. **(C)** Lipidomics analysis on WT and DAGL KD Neuro-2a populations treated *in situ* with vehicle or DH376 (100 nM, serum-free, 2 h). Lipid abundance was normalized for the amount of protein. Data is expressed as % of vehicle [mean ± SEM (WT: *n* = 8, KD: *n* = 4/2), *t*-test: ^∗^*p* < 0.05, ^∗∗^*p* < 0.01, ^∗∗∗^*p* < 0.001]. **(D)** DAGL KD efficiency was assessed by chemical proteomics on WT and KD Neuro-2a cells using probes MB108 and FP-biotin (10 μM each, 30 min, 37°C). Data is expressed as % of WT-vehicle [mean ± SEM (*n* = 4), *t*-test with Holm–Sidak multiple comparison correction: ^∗^*p* < 0.05, ^∗∗^*p* < 0.01, ^∗∗∗^*p* < 0.001].

To investigate which DAGL isoform is responsible for 2-AG production in Neuro-2a cells, we used a genetic approach as no subtype-specific DAGL inhibitors are currently available. Of note, single cell heterogeneity (in 2-AG production and neurite outgrowth) prevented the unequivocal analysis of single cell clone knockouts (Supplementary Figure S1). Therefore, disruption of DAGLα and DAGLβ genes was performed by three sequential rounds of transfection of Cas9 and single guide RNA’s (sgRNA) in Neuro-2a cell populations (Supplementary Figures S2A–C). This yielded three Neuro-2a knockdown (KD) populations: DAGLα KD, DAGLβ KD, and DAGLα-β KD. DAGLα and DAGLβ activity in these cell populations was measured using activity-based protein profiling (ABPP) to determine the efficiency of the genetic disruption. ABPP is a chemical proteomic method that uses chemical probes (e.g., fluorophosphonates (FP) or β-lactones) to assess the functional state of various enzymes, here DAGLα and DAGLβ, in native biological systems. When coupled to fluorescent reporter groups, activity-based probes (ABPs) enable visualization and quantification of enzymatic activity in complex proteomes by sodium dodecyl sulfate-polyacrylamide gel electrophoresis (SDS-PAGE) and in-gel fluorescence scanning. When coupled to a biotin reporter group, ABPs enable affinity enrichment and identification of enzyme activities by mass spectrometry (MS)-based proteomics. Gel-based ABPP with a fluorescent FP-probe (FP-TAMRA) and β-lactone probe MB064 showed a reduction (>70%) of active DAGLβ in the DAGLβ KD and DAGLα-β KD populations (Figures 3A,B), without affecting other serine hydrolase activities. LC-MS-based chemical proteomics confirmed these findings (Figure 3D). Of note, no DAGLα activity was observed in either of the populations, including WT Neuro-2a (Figures 3A,B). The residual DAGLβ activity can be explained by a transfection efficiency below 100% and by insertion or deletion of a full codon upon Cas9-mediated DNA modification, thus preventing the frameshift that generally results in an early stop-codon.

Next, the 2-AG levels of the genetically modified Neuro-2a populations were quantified using LC-MS. Surprisingly, despite a >70% reduction in levels of active DAGLβ and no detectable DAGLα activity, the 2-AG levels in the DAGLα KD, DAGLβ KD or double DAGLα-β KD populations were not different from WT populations (Figure 3C). To test whether the 2-AG production in the KD populations was still sensitive to DH376 treatment, cells were incubated *in situ* (live cells) with DH376 (100 nM, 2 h). Lipidomics analysis on these samples revealed that DH376 again reduced cellular levels of both 2-AG and AA by 50% in all populations (Figure 3C). Of note, a small but significant increase in AEA levels was detected in the double DAGLα-β KD populations (Figure 3C), which could be due to increased levels of active ABHD4 (Figure 3D). Taken together, these data suggest that residual DAGLβ activity may be responsible for generating the entire pool of 2-AG, which seems unlikely, or that an alternative, unidentified enzyme, which is sensitive to DH376, contributes to 2-AG production in Neuro-2a cells.

**FIGURE 4:**
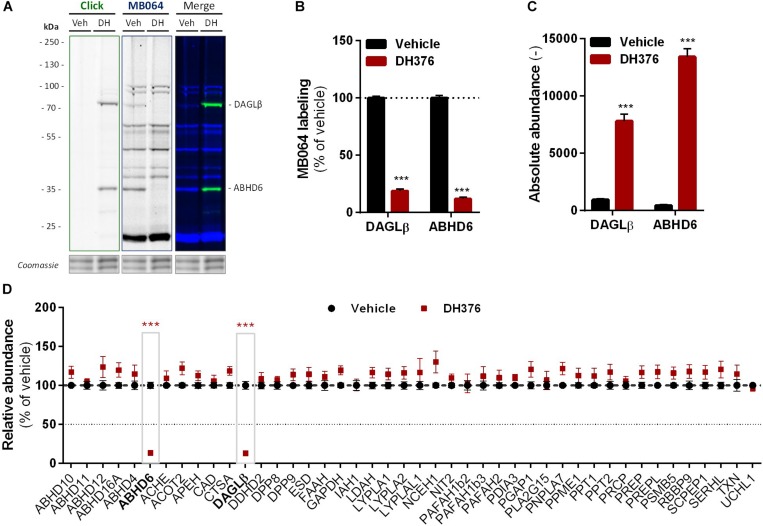
DH376 targets DAGLβ and ABHD6 in Neuro-2a. Neuro-2a cells were treated *in situ* with vehicle or DH376 (100 nM, serum-free, 2 h) to investigate the DH376 interaction profile. **(A,B)** *In situ* DH376 targets were visualized by gel-based ABPP after conjugation of DH376 to Cy5-azide (5 μM, 60 min, rt) or with probe MB064 (2 μM, 20 min, rt). Coomassie served as protein loading control. **(B)** Probe labeling was quantified and normalized for protein loading. Data is expressed as % of vehicle [mean ± SEM (Veh *n* = 9, DH376 *n* = 3), *t*-test: ^∗∗∗^*p* < 0.001]. **(C)** Chemical proteomics enabled DH376 target identification. Lysates of *in situ* DH376 treated Neuro-2a cells were conjugated to biotin-azide (40 μM, 60 min, 37°C). Vehicle treated samples served as a negative control. Data is expressed as absolute abundance [mean ± SEM (*n* = 4), *t*-test: ^∗∗∗^*p* < 0.001]. **(D)** Competitive proteomics validated ABHD6 and DAGLβ as DH376 targets in *in situ* treated Neuro-2a cells, using probes MB108 and FP-biotin (10 μM each, 30 min, 37°C). Data is expressed as % of WT-Vehicle [mean ± SEM (*n* = 4), *t*-test with Holm–Sidak multiple comparison correction: ^∗∗∗^*p* < 0.001].

To identify all 2-AG producing enzymes targeted by DH376 in Neuro-2a, we leveraged a chemical proteomics strategy in which the alkyne moiety of DH376 served as a ligation handle to introduce a reporter group via a copper(I)-catalyzed azide-alkyne cycloaddition (“click” chemistry) (Rostovtsev et al., 2002). Neuro-2a cells were incubated with DH376, lysed and the covalently bound inhibitor-target complexes were conjugated to Cy5-azide and visualized by SDS-PAGE and in-gel fluorescence scanning (Figures 4A,B). Apart from DAGLβ, one other fluorescent band with a molecular weight of ∼35 kDa was detected. Competitive ABPP using MB064 and FP-TAMRA suggested that this activity could be ABHD6, which was previously also reported as an off-target of DH376 (Ogasawara et al., 2016; van Rooden et al., 2018). To confirm the identity of this protein expressed by Neuro-2a cells, chemical proteomics was employed (Figure 4C). DAGLβ and ABHD6 were identified as the only targets of DH376 in Neuro-2a cells (Figure 4D). Thus, these data suggested that ABHD6 could be responsible for 2-AG production in conjunction with DAGLβ.

**FIGURE 5:**
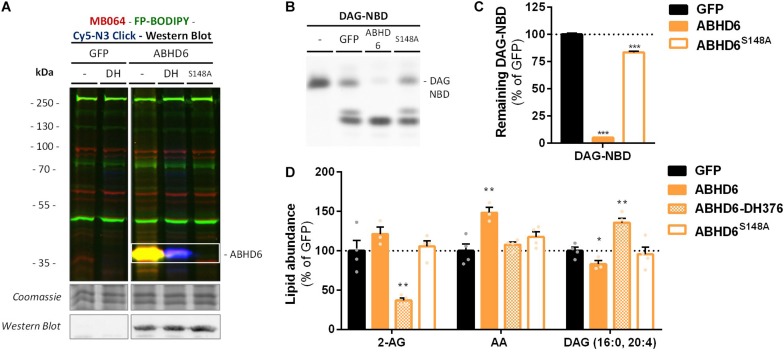
Recombinant ABHD6 possesses DAG-lipase activity *in vitro* and *in situ*. HEK293-T cells were transiently transfected with GFP, ABHD6 or its catalytically inactive serine mutant (S148A) and treated *in situ* with vehicle or DH376 (DH, 1 μM, 2 h, serum-free). **(A)** Protein activity and expression was confirmed by gel-based ABPP and western blot. Samples were subsequently incubated with probes MB064 (red; 500 nM, 10 min, rt), FP-BODIPY (green; 500 nM, 10 min, rt), and Cy5-azide click mix (blue; 2.5 μM, 30 min, rt). Coomassie served as a protein loading control. Western blot with mouse-anti-FLAG (1:2500, 45 min, rt) verified expression of the catalytically inactive protein. **(B,C)** Whole cell lysates were incubated with DAG-NBD (5 μM, 30 min, 37°C), lipids were extracted and analyzed by HPTLC. **(C)** DAG hydrolysis was quantified and expressed as % of GFP [mean ± SEM (*n* = 3), *t*-test: ^∗∗∗^*p* < 0.001]. **(D)** Lipid abundance of transfected and *in situ* treated cells was measured and normalized to the amount of protein. Data is expressed as % of GFP-Vehicle [mean ± SEM (*n* = 4), *t*-test: ^∗^*p* < 0.05, ^∗∗^*p* < 0.01].

α,β-Hydrolase domain containing protein 6 is known to hydrolyze additional lipids than 2-AG, including lysophosphatidyl species (Thomas et al., 2013) and bis(monoacylglycero) phosphate (Pribasnig et al., 2015). To determine if ABHD6 can use DAG as a substrate, a DAG hydrolysis assay was developed based on fluorescent 1-nitrobenzoxadiazole-decanoyl-2-decanoyl-*sn*-glycerol (NBD-DAG) substrate. Lysates from HEK293-T cells overexpressing recombinant human ABHD6 (Figure 5A) or its catalytically inactive mutant (ABHD6^S148A^) as a negative control, were incubated with NBD-DAG and analyzed by HPTLC. Lysates from HEK293-T cells expressing DAGLα or its catalytically inactive mutant (DAGLα^S472A^) served as positive and negative controls, respectively (Supplementary Figure S3A). Both DAGLα and ABHD6 exhibited DAG-lipase activity as their overexpression resulted in the hydrolysis of NBD-DAG, whereas their mutants did not (Figures 5B,C and Supplementary Figures S3B,C), thereby showing that ABHD6 hydrolyzes the *sn*-1 ester bond of an *sn*-1-acyl-2-decanoyl-glycerol. Of note, the NBD-DAG hydrolysis measured in HEK293-T cells expressing GFP reflects the conversion of the substrate by endogenous hydrolases, including ABHD6, and to a lesser extent DAGLβ.

In view of the abundant ABHD6 activity in different brain regions (Baggelaar et al., 2017), we tested whether ABHD6 contributes to DAG hydrolysis in mouse brain. However, no reduction in DAG-NBD hydrolysis was observed in mouse brain lysates treated with the selective ABHD6 inhibitor KT182 or by comparing lysates from *Abhd6^–/–^* mouse brain and WT controls (Supplementary Figure S4). Of note, mice could not be age-matched which may affect expression levels. However, aside from the loss of ABHD6 in knockout tissue, no evident discrepancies were observed in the lipase activity profiles (Supplementary Figure S4A).

Next, we determined if ABHD6 could also hydrolyze endogenous DAGs in intact cells. To this end, recombinant ABHD6 was overexpressed in HEK293-T cells (DAGLα as positive control and the catalytically inactive mutants as negative controls) and endogenous DAG (16:0, 20:4) levels were determined by targeted lipidomics (Figure 5D and Supplementary Figure S3D). Both ABHD6 and DAGLα overexpression reduced the levels of DAG (16:0, 20:4), whereas overexpression of the catalytically inactive mutants had no effect on the DAG-levels. DH376 treatment of the transfected cells prevented the reduction in DAG levels and in fact led to an increase in this lipid species, indicating that this DAG species serves also as an endogenous substrate for DAGLα/ABHD6. Overexpression of ABHD6 did not affect 2-AG levels, in line with the dual MAG/DAG-lipase character of ABHD6, whereas its final product AA was increased (Figure 5D). In summary, these data indicate that ABHD6 acts as a DAG/MAG-lipase using DAG (16:0, 20:4) as an endogenous substrate.

**FIGURE 6:**
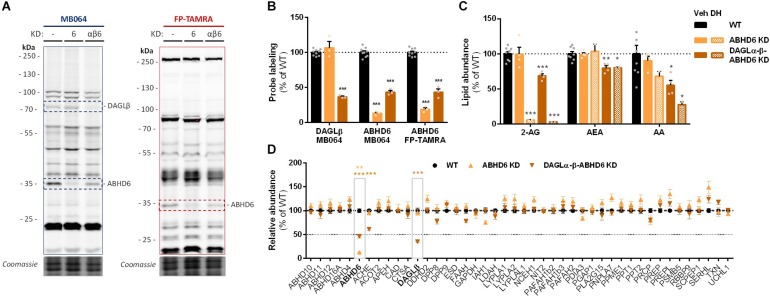
Neuro-2a DAGLα–β-ABHD6 triple KD populations have decreased 2-AG levels. **(A,B)** ABHD6 and DAGLα–β-ABHD6 KD populations were analyzed by gel-based ABPP using probes MB064 (2 μM) and FP-TAMRA (500 nM) (20 min, rt). Coomassie served as a protein loading control. **(B)** Probe labeling was normalized for protein loading. Data is expressed as % of vehicle [mean ± SEM (WT: *n* = 9, KD: *n* = 3), *t*-test: ^∗∗∗^*p* < 0.001]. **(C)** Lipidomics analysis on WT and ABHD6 KD Neuro-2a populations treated *in situ* with vehicle or DH376 (100 nM, serum-free, 2 h). Lipid abundance was normalized for the amount of protein. Data is expressed as % of vehicle [mean ± SEM (WT: *n* = 8, KD: *n* = 4/2), *t*-test: ^∗^*p* < 0.05, ^∗∗^*p* < 0.01, ^∗∗∗^*p* < 0.001]. **(D)** DAGL and ABHD6 KD efficiency was assessed by chemical proteomics on WT and KD Neuro-2a populations using probes MB108 and FP-biotin (10 μM each, 30 min, 37°C). Data is expressed as % of WT-vehicle [mean ± SEM (*n* = 4), *t*-test with Holm–Sidak multiple comparison correction: ^∗∗^*p* < 0.01, ^∗∗∗^*p* < 0.001].

To check whether endogenous cellular levels of 2-AG and AA are controlled by ABHD6, a Neuro-2a ABHD6 KD population and a triple DAGLα-β-ABHD6 KD population were generated (Figure 6A and Supplementary Figures S2D–F). ABHD6 KD had no effects on 2-AG or AA levels (Figure 6C), which suggests that DAGLβ and ABHD6 activities can compensate for each other. The KD efficiency at the protein level was reduced in the triple DAGLα-DAGLβ-ABHD6 KD as compared to the single KDs as determined by gel-based ABPP (Figures 6A,B) and chemical proteomics (Figure 6D). We noticed that 40–50% of active ABHD6 and 30–40% active DAGLβ remained. In line with the reduced ABHD6 and DAGL activity in the triple KD, both 2-AG and AA levels were reduced by approximately 30%.

**FIGURE 7:**
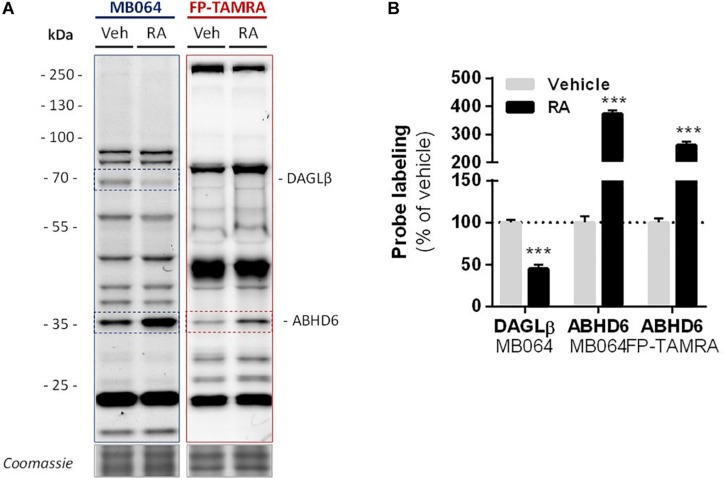
Levels of active DAGLβ decreased while active ABHD6 levels increased during RA-induced differentiation of Neuro-2a. Neuro-2a cells were stimulated by *in situ* treatment with retinoic acid (RA, 50 μM, 2% serum, 72 h). **(A)** Whole lysates of vehicle or RA stimulated cells were analyzed by gel-based ABPP using activity-based probes MB064 (2 μM) or FP-TAMRA (500 nM) (20 min, rt). Coomassie served as a protein loading control. **(B)** Probe labeling was normalized to loading control. Data is expressed as % of vehicle [mean ± SEM (*n* = 3), *t*-test, ^∗∗∗^*p* < 0.001].

Finally, in light of the finding that ABHD6 can act as a DAG lipase, the DAGLβ and ABHD6 activity in Neuro-2a cells during RA-induced differentiation was mapped by gel-based ABPP (Figure 7A). A threefold increase in the amount of active ABHD6 was observed in differentiated Neuro-2a cells, whereas the amount of active DAGLβ was decreased (Figure 7B). However, inhibition of ABHD6 by the ABHD6 inhibitor KT182 did not abolish the 2-AG increase during RA-induced differentiation (Supplementary Figure S5). As the dual ABHD6/DAGL inhibitor DH376 inhibitor did reduce 2-AG levels and differentiation (Figure 2), these data suggest that the RA-induced 2-AG production (Figure 1B) in Neuro-2a cells is due to combined DAGLβ and ABHD6 activity.

## Discussion

In the present study, we extend our understanding of ABHD6 to a dual DAG/MAG-lipase that produces 2-AG. We also show that ABHD6 activity is involved in 2-AG production in Neuro-2a cells during RA-induced differentiation. Previously, it was shown that the RA-induced neurite outgrowth is a CB1R dependent process (Jung et al., 2011). The dual ABDH6/DAGL inhibitor DH376 reduced RA-induced Neuro-2a differentiation, thereby supporting the notion that 2-AG is involved in this process. Nevertheless, it is likely that other lipid mediators also play a role, because DH376 did not abolish neurite outgrowth and AEA and AA levels were dramatically increased during differentiation.

α,β-hydrolase domain containing protein 6 is a highly conserved metabolic hydrolase that belongs to the serine hydrolase family (Navia-Paldanius et al., 2012), mainly known as a 2-AG hydrolytic enzyme, but its substrates also include other monoacylglycerols (Navia-Paldanius et al., 2012), as well as various lysophosphatidyl species (Thomas et al., 2013) and bis(monoacylglycero)phosphate (Pribasnig et al., 2015). Using biochemical, genetic, and pharmacological approaches, we expanded the substrate scope of ABHD6 toward DAGs. Considering both MAG and DAG hydrolysis are mediated by the same catalytic serine (S148) (Figure 5; Navia-Paldanius et al., 2012), the respective MAG- and DAG-activities are therefore likely to be driven by relative substrate and product concentrations rather than by regulatory mechanisms. Our data indicate that 2-AG and AA are both produced by DAGL and ABHD6 during neuronal differentiation.

Although ABHD6 accounts for only 4% of 2-AG hydrolysis in mouse brain homogenates (Blankman et al., 2007), it is an important factor of MAG hydrolysis in specific cell- or tissues types, for example, in BV-2 cells where MAGL is lacking (Muccioli et al., 2007). In a similar fashion, the contribution of ABHD6 to DAG hydrolysis and 2-AG biosynthesis in the whole brain is likely to be limited, but it may be more pronounced in specific cell-types and physiological processes in the absence of DAGLα or DAGLβ. In the adult central nervous system, the postsynaptic localization of ABHD6 (Marrs et al., 2010) would indeed allow for retrograde endocannabinoid signaling. In the young brain, ABHD6 mRNA is found detected in progenitor cells (Gokce et al., 2016), which may provide an additional mechanism for cell-autonomous eCB-signaling (Goncalves et al., 2008). Notably, the localization of MAGL is complementary to that of ABHD6 (Straiker et al., 2009) with MAGL mainly localized at the presynaptic site. In a global MAGL KO mouse model no compensatory effects were observed by ABHD6 (Taschler et al., 2011). Similarly, ABHD6 deletion using antisense oligonucleotides did not affect MAGL activity in peripheral tissue (Thomas et al., 2013). Taken together, this suggests that, despite both being MAGLs, the physiological functions of ABHD6 and MAGL may be distinct.

Recent studies suggest that ABHD6 inhibitor have promising therapeutic efficacy in several preclinical mouse models of devastating diseases (Cao et al., 2019), such as metabolic syndrome (Thomas et al., 2013), chronic inflammation (Alhouayek et al., 2013), diabetes (Zhao et al., 2014), including traumatic brain injury (Tchantchou and Zhang, 2013), multiple sclerosis (Manterola et al., 2018), and epilepsy (Naydenov et al., 2014). Thus, it is important to further study the impact of the DAG lipase activity of ABHD6 on these pathophysiological processes to help develop novel therapeutics based on inhibiting ABHD6 activity.

## Conclusion

We discovered that ABHD6 hydrolyzes DAG, thereby contributing to the production of the bioactive signaling lipid 2-AG during RA-induced differentiation of Neuro-2a cells. Our finding is supported by biochemical, genetic and pharmacological evidence. First, genetic knockdown of both DAGLα and DAGLβ in Neuro-2a cells had no effect on cellular 2-AG levels. Second, the dual DAGL/ABHD6 inhibitor DH376 abolished cellular 2-AG levels. Third, ABHD6 catalyzed the degradation of a fluorescent DAG substrate. Fourth, overexpression of ABHD6, but not its catalytically inactive mutant, reduced endogenous DAG (16:0, 20:4) levels, which was inhibited by DH376. Fifth, cellular 2-AG levels decreased upon triple KD of DAGLα-β-ABHD6 in Neuro-2a. These data extend the role of ABHD6 from a MAG lipase to a DAG lipase and suggest a potential role for ABHD6 in neuronal differentiation.

## Data Availability Statement

The raw data supporting the conclusion of this manuscript will be made available by the authors, without undue reservation, to any qualified researcher.

## Ethics Statement

The animal study was reviewed and approved by the mice were housed in a pathogenic-free facility in accordance with the National Institutes of Health; the Institutional Animal Care and Use Committee at the University of Washington approved all experiments.

## Author Contributions

AE and MS contributed to the conception and design of the study. AE carried out the experiments and analyzed the data. XD and VK measured and analyzed the lipidomics experiments. AB and BF assisted with the LC/MS measurements and data analysis of chemical proteomic experiments. TW assisted in sgRNA design and cloning. TW and AS assisted in experiments. NS and SS provided the mouse brain tissues from WT and *Abhd6^–/–^* mice. HD cloned and prepared the plasmids for recombinant expression. AE and MS wrote the manuscript. All authors contributed to the manuscript revision, read, and approved the submitted version.

## Conflict of Interest

The authors declare that the research was conducted in the absence of any commercial or financial relationships that could be construed as a potential conflict of interest.

## Footnotes

1 http://chopchop.cbu.uib.no
