# Supplementary material for: Identification of α,β-Hydrolase Domain Containing Protein 6 as a Diacylglycerol Lipase in Neuro-2a Cells
Source: Front Mol Neurosci. 2019 Nov 26;12:286. doi: 10.3389/fnmol.2019.00286 (PMC6901982; doi:10.3389/fnmol.2019.00286)
Supplement: Supplementary file 1 [file Image_1.pdf]

Supplementary materials accompanying

# Identification of α,β-hydrolase domain containing protein 6 as a diacylglycerol lipase in Neuro-2a cells

**Annelot. C. M. van Esbroeck[1*], Vasudev Kantae[1,2*], Xinyu Di[2], Tom van der Wel[1], Hans den Dulk[1], Anna F. Stevens[1], Simar Singh[4,5], Alexander T. Bakker[1], Bogdan I. Florea[3], Nephi Stella[4,5], Herman S. Overkleeft[3], Thomas Hankemeier[2] and Mario van der Stelt[1]**

[1] Department of Molecular Physiology, Leiden Institute of Science, Leiden University, Leiden, The Netherlands

[2] Department of Systems Biomedicine and Pharmacology, Leiden Academic Centre for Drug Research, Leiden University, Leiden, The Netherlands.

[3] Department of Bio-Organic Synthesis, Leiden Institute of Chemistry, Leiden University, Leiden, The Netherlands.

[4] Department of Pharmacology, University of Washington, Seattle, WA, USA

[5] Department of Psychiatry and Behavioral Sciences, University of Washington, Seattle, WA, USA

* Authors contributed equally.

**Correspondence:** Prof. dr. Mario van der Stelt, m.van.der.stelt@lic.leidenuniv.nl

# 1 Supplementary Figures

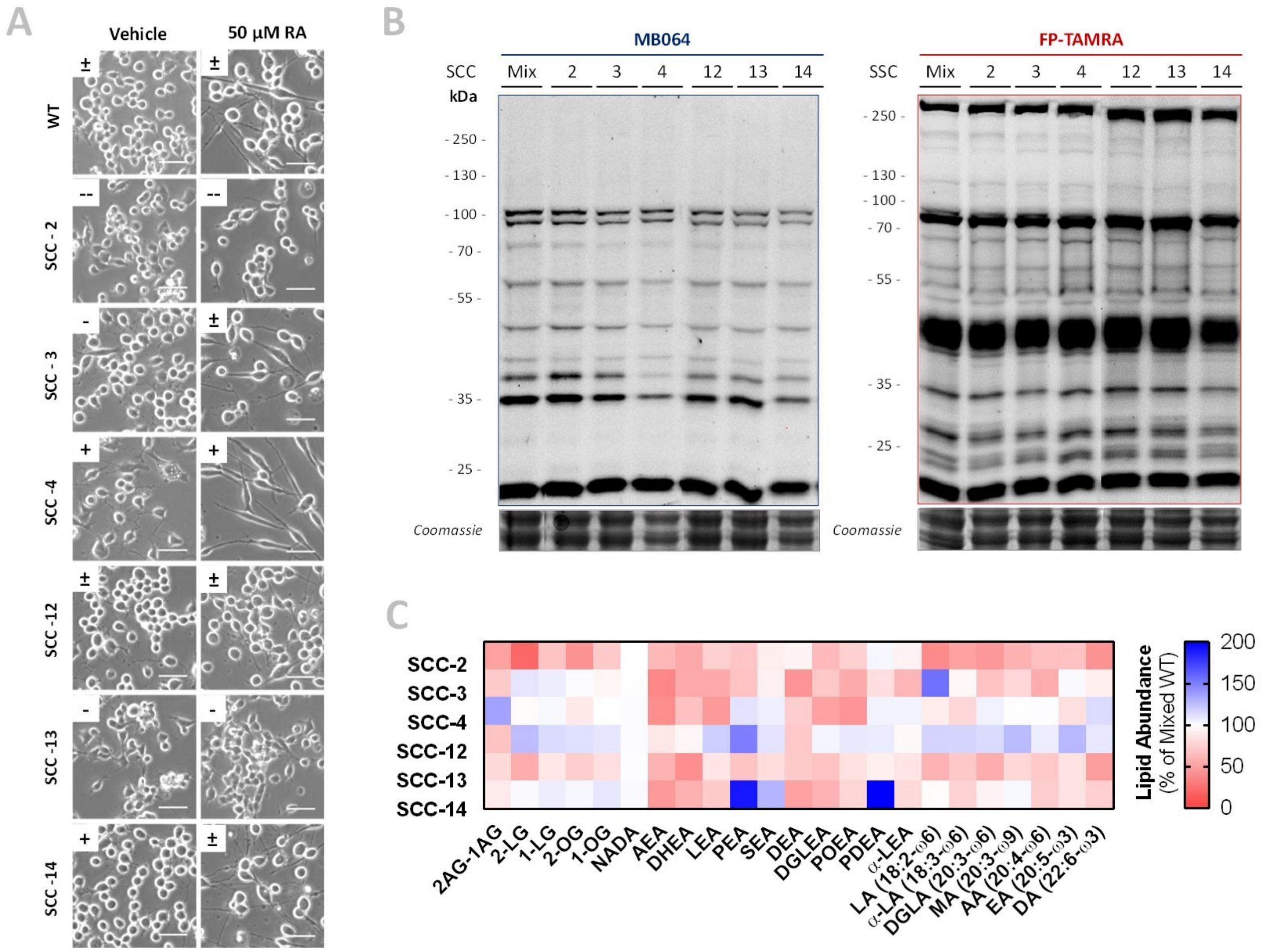


**Figure S1 | Neuro-2a single cell clones have diverging phenotypes and lipid profiles.**
(**A**) Neuro-2a and Neuro-2a single cell clones (SCCs) show a diverging extent of neurite outgrowth upon treatment with retinoic acid (50 µM, 0.1% DMSO, 2% serum, 48 hrs.). -/±/+ indicate the state of neurite outgrowth as compared to the mixed culture under the same conditions. Scale bar: 50 µm. (**B**) WT Neuro-2a and six different WT Neuro-2a SCCsnwere analyzed by gel-based ABPP using probes MB064 (250 nM) and FP-TAMRA (500 nM) (20 min, rt). Coomassie served as a protein loading control. (**C**) Heat map summary of lipidomics performed on Neuro-2a and Neuro-2a SSCs revealed variation in lipid abundance between clones. Lipid abundance was normalized to cell number and data is expressed as % of WT (n=3).

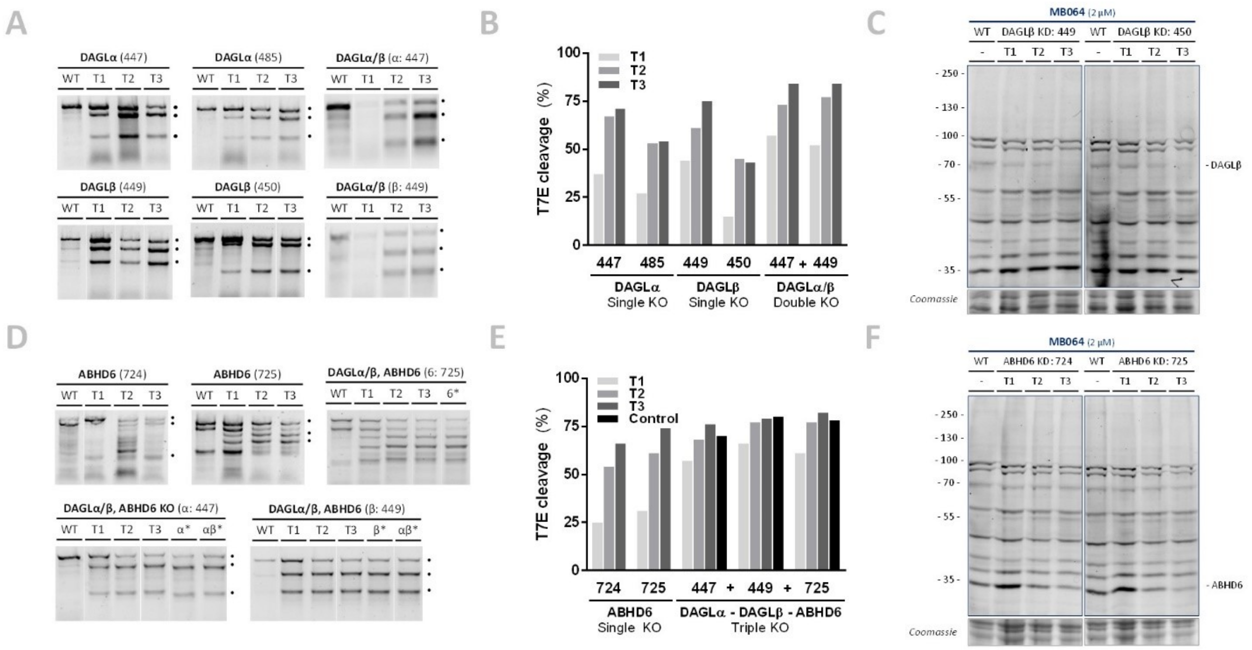


**Figure S2 | Efficiency of CRISPR/Cas9-mediated knockdown in Neuro-2a.** Knockdown populations were generated by three sequential transfections (T1-T3) with Cas9 and two different separate guides for each target. Most efficient guides were used to generate double and triple knockdowns. (**A**-**B**, **D**-**E**) Knockdown efficiency is determined by a T7E1 assay on genomic DNA after each round of transfection and quantified for DAGL (**A**-**B**) and ABHD6 (**D**-**E**) knockdowns. (**C**, **F**) ABPP analysis of knockdown efficiency. After each transfection, whole lysates were incubated with MB064 (2 µM, 20 min, rt) and analyzed by SDS-PAGE.

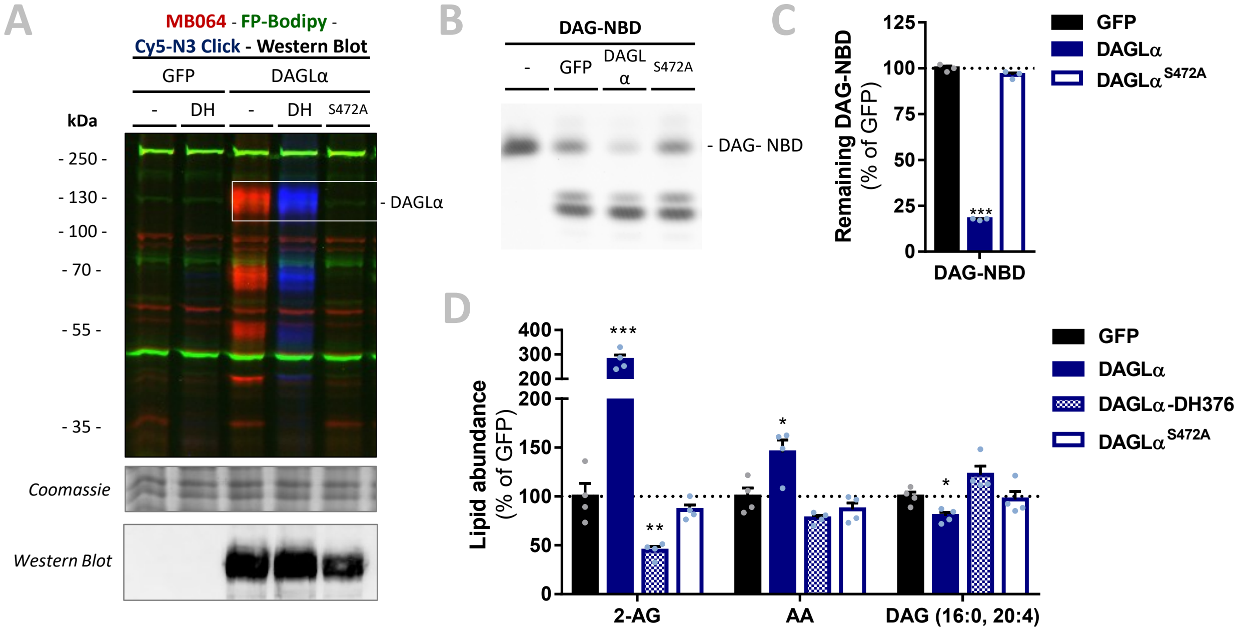


**Figure S3 | Recombinant DAGLα possesses DAG-lipase activity *in vitro* and *in situ*.** HEK293-T cells were transiently transfected with GFP, DAGLα or its catalytically inactive serine mutant (S472A) and treated *in situ* with vehicle or DH376 (DH, 1 µM, 2 hrs., serum-free). (**A**) Protein activity and expression were confirmed by gel-based ABPP and western blot. Samples were subsequently incubated with probes MB064 (500 nM, 10 min, rt), FP-BODIPY (500 nM, 10 min, rt), and Cy5-azide click mix (2.5 µM, 30 min, rt). Coomassie served as a protein loading control. Western blot with mouse-anti-FLAG (1:2500, 45 min, rt) verified expression of the catalytically inactive protein. (**B**-**C**) Whole lysates were incubated with DAG-NBD (5 µM, 30 min, 37 °C). Lipids were extracted and analyzed by HPTLC (n=3). DAG hydrolysis was quantified and expressed as % of GFP (mean ± SEM (n=3), *t*-test: *** $p < 0.001$). (**D**) Lipid abundance of transfected and *in situ* treated cells was measured and normalized to the amount of protein (n=4). Data is expressed as % of GFP-Vehicle (mean ± SEM (n=4), *t*-test: * $p < 0.05$, ** $p < 0.01$, *** $p < 0.001$).

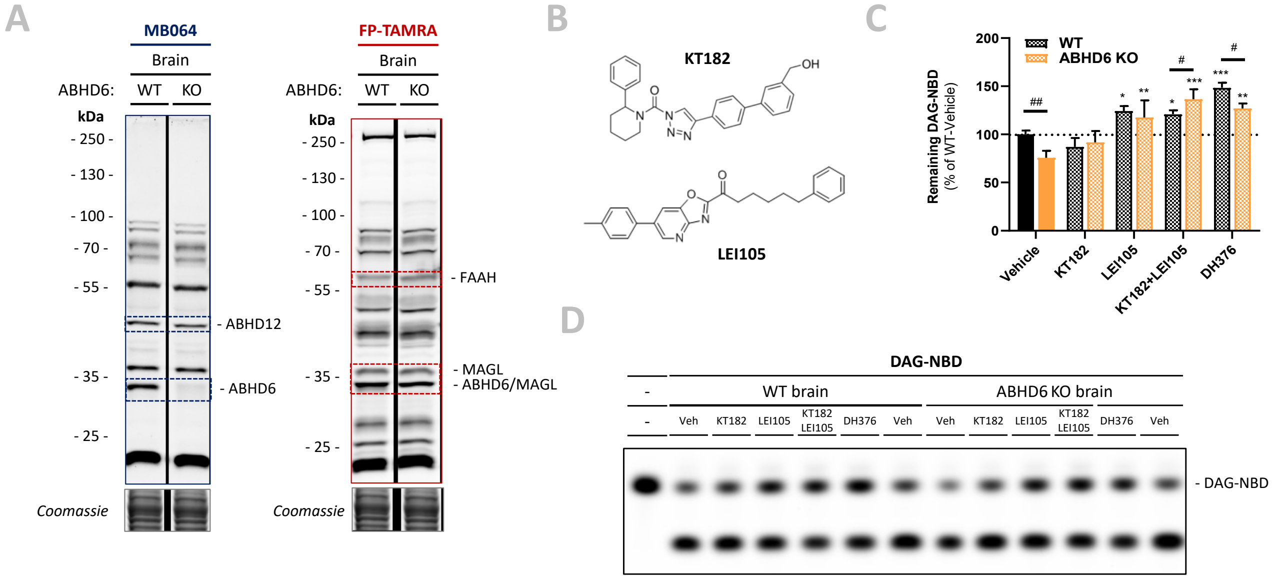

**Figure S4 | DAG-NBD hydrolysis in mouse brain homogenates is not affected by KO or inhibition of ABHD6 alone.** Mouse brain homogenates were prepared from mice with a WT or *Abhd6*$^{-/-}$ background. (**A**) ABHD6 KO was verified by gel-based ABBP. Mouse brain homogenates were incubated with probe MB064 (250 nM, 20 min, rt) or FP-TAMRA (500 nM, 20 min, rt). Coomassie served as a protein loading control. (**B**-**D**) Brain homogenates were incubated with vehicle or inhibitors of ABHD6 (KT182, (**B**)), DAGL (LEI105 (**B**)) or dual DAGL-ABHD6 inhibitor DH376 (100 nM 30 min, 37 °C) prior to incubation with DAG-NBD (10 µM, 15 min, 37 °C). (**D**) Lipids were extracted and analyzed by HPTLC. (**C**) DAG hydrolysis was quantified and expressed as % of WT-Vehicle (mean ± SEM (n=3), *t*-test Inhibitor versus Vehicle: * p < 0.05, ** p < 0.01, *** p < 0.001; WT versus KO: # p < 0.05, ## p < 0.01).

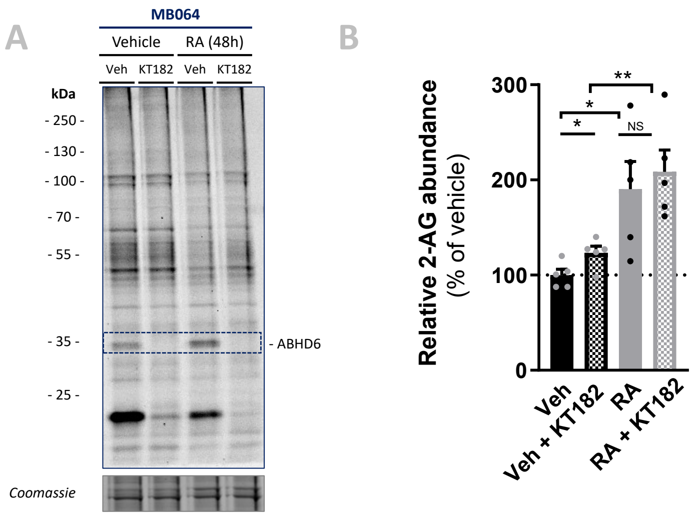


**Figure S5 | ABHD6 inhibitor KT182 does not abolish increased 2-AG levels upon RA-induced differentiation.** Neuro-2a cells were stimulated by *in situ* treatment with retinoic acid (RA, 50 µM, 2% serum, 48 h) with or without co-treatment with ABHD6 inhibitor KT182 (100 nM). (**A**) Whole lysates of vehicle or RA stimulated cells were analyzed by gel-based ABPP using activity-based probe MB064 (2 µM, 20 min, rt). Coomassie served as a protein loading control. (**B**) Lipidomics analysis on (co-)treated Neuro-2a (48 hrs). Lipid abundance was normalized to protein concentration. Data is expressed as % of vehicle (mean ± SEM (n=5), *t*-test: * $p < 0.05$, ** $p < 0.01$, *** $p< 0.001$, NS not significant).

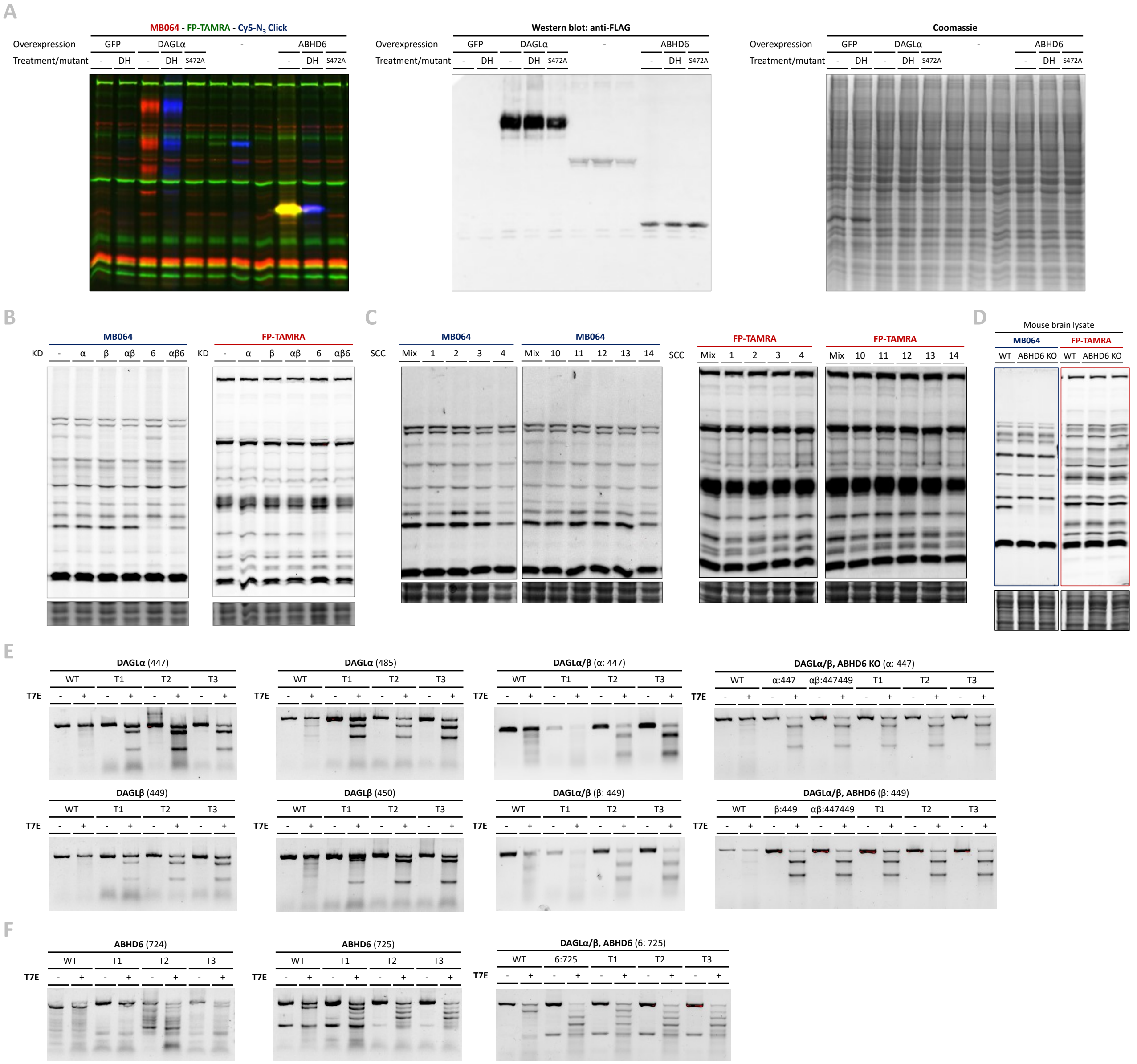


**Figure S6 | Full images of cropped/combined gels and blots.** Experimental details are described in the original figure captions, relevant lanes are annotated. (**A**) Figure 5A; (**B**) Figure 6A; (**C**) Figure S1B; (**D**) Figure S4A; (**E**) Figure S2A, D; (**F**) Figure S2D.

## 2 Supplementary files

**Supplementary Table 1 - Peptide Lists (Esbroeck et al., Frontiers, 2019).xlsx**

**Supplementary Table 1 |** Peptide lists of LC-MS based chemical proteomics / activity-based proteomics experiments.
